# Universal health coverage must include older people

**DOI:** 10.2471/BLT.17.204214

**Published:** 2018-01-01

**Authors:** Ritu Sadana, Agnes Soucat, John Beard

**Affiliations:** a Department of Ageing and Life Course, World Health Organization, avenue Appia 20, 1211 Geneva 27, Switzerland.; b Health Systems Governance and Financing, World Health Organization, Geneva, Switzerland.; Correspondence to Ritu Sadana (email: sadanar@who.int).

The health sustainable development goal (SDG3) aims to “ensure healthy lives and promote wellbeing for all, at all ages.” One of its core targets is to achieve universal health coverage (UHC). However, older adults require different approaches to health care and are often less able to pay for these services; therefore, health systems will need to be realigned significantly to meet these targets.^1^ The World Health Organization (WHO) *Global strategy and action plan on ageing and health*^2^ provides a political mandate for action to enable this transformation.

The strategy focuses on strengthening health and long-term care systems at local and national levels to cover the needs of older adults through strengthening national policy, combating ageism, generating new evidence and supportive tools and creating more age-friendly environments. Sustainable financing, a key concern of policy-makers,^3^ must also be addressed, as ageing societies are likely to present significant challenges: ageing health workforce, higher disease burden^4^ and increased demand for services and for people who provide care.

These challenges call for three interlinked strategies to make health systems fit for purpose and context: strengthening the systems’ foundations, reforming institutions and fostering transformation. Implementing these changes demands shifting away from disease-oriented financing, resources and management, towards a person-centred agenda that values health gain.^5^ Implementing these policies and actions will advance healthy ageing and increase older adults’ capacities and ability to function where they live, replacing the belief that older people only need specialized medical treatments for each disease or condition.

Although every country is different, steps can be taken towards UHC that is more inclusive of older adults. 

First, countries need to foster better integration between health and social care to improve and maintain older adults’ physical and cognitive capacities. Older people who benefit from coordinated chronic care that is guided by comprehensive assessments report more satisfaction and experience fewer emergency referrals than when they receive services that treat individual conditions independently.^6^ Reforms in Thailand have fostered better integration between health and social care,^7^ and those in Chile have added services that improve and maintain physical and cognitive capacities.^8^ Ensuring essential medicines and assistive devices and adapting environments of those who are care dependent, at home or in neighbourhoods, increases the number of older adults who function better and have more meaningful lives.^3^ Hong Kong, China, offers a case study on documenting the comprehensive needs of older adults. This approach combines medical and social services and offers financial incentives to older adults to use community centres to address geriatric syndromes and conditions, from dementia to chewing, visual and hearing difficulties.^9^WHO’s guidelines on *Integrated care for older people,*^10^ promote six community-level interventions to manage the decline of physical and mental capacities; the guidelines also include interventions to support caregivers.

Second, policy-makers must negotiate ways to reduce costs and increase risk sharing. The WHO Survey on Global Ageing and Adult Health in China, Ghana, India, Mexico, the Russian Federation and South Africa, documents that households with people older than 50 years experienced greater financial burden due to health costs,^11^ compared to households without older people, including higher rates of impoverishment, catastrophic health expenditures and borrowing of money to pay for health services. For the same countries, health insurance generally increased access to care, but gave insufficient protection against financial hardship.^12^ For equitable access and financial protection, financing services should pool together all people and not discriminate by age, employment, residential or health status. With the world’s oldest population, Japan offers experience in 50 years of political commitment, progressive financing mechanisms, coverage of long-term care services and strategic purchasing of services, drugs and devices. These actions can result in cost savings, reduce cost escalation, enhance gender equity and foster innovations towards UHC that is inclusive of older persons.^13^

Finally, achieving UHC requires countries to pay attention to health inequities. Differences in functioning during the second half of life reflect the cumulative impact of many social and environmental determinants.^14^ Interventions must assess impact on all ages, gender and socioeconomic groups. The government-supported Longitudinal Ageing Study in India illustrates a comprehensive situation assessment, which will enable analysis and planning within each Indian state and territory for the next 25 years.

To progress towards UHC, policy-makers must address competing priorities for different population groups, services and financing mechanisms. The commitment of the Senegalese Ministry of Health and Social Action to review its plan covering individuals 60 years and older, conduct further analyses in rural areas and develop a new national strategy towards healthy ageing shows that actions can be informed by policy dialogues across government sectors and be inclusive of civil society, so that on the road to UHC, no one is left behind.
